# Sugarcane Bagasse-Derived Biochar-Enabled Microbial Fuel Cell for Concurrent Bioelectrochemical Energy Recovery and Wastewater Remediation

**DOI:** 10.3390/biomimetics11070443

**Published:** 2026-06-24

**Authors:** Seyedrahman Djafaripetroudy, Mabel Lagla-Molina, Alex Guambo-Galarza, Norma Erazo, Magdy Echeverría, Angel Ordóñez

**Affiliations:** 1 Natural Resources Management Faculty, Lakehead University, 955 Oliver Rd., Thunder Bay, ON P7B 5E1, Canada; 2 Biorefinery Research Institute (BRI), Lakehead University, 1294 Balmoral St., Thunder Bay, ON P7B 5Z5, Canada; 3 Independent Research, Riobamba 060107, Ecuador; jhoselinlagla380@gmail.com; 4 Research and Development Group for the Environment and Climate Change (GIDAC), Escuela Superior Politécnica de Chimborazo (ESPOCH), Riobamba 060106, Ecuador; aguambo@espoch.edu.ec (A.G.-G.); norma.erazo@espoch.edu.ec (N.E.); m_echeverria@espoch.edu.ec (M.E.); angel.ordoniez@espoch.edu.ec (A.O.)

**Keywords:** bioelectrochemical, biomimetics, energy recovery, food and vegetable leachate, microbial fuel cell, sugarcane bagasse, wastewater remediation

## Abstract

Microbial fuel cells (MFCs) are emerging as biomimetic bioelectrochemical systems that emulate naturally occurring microbial electron-transfer pathways for stimulus bioenergy generation and wastewater remediation. In this study, food–vegetable leachate (FVL) and sugarcane bagasse-derived biol were evaluated in combination with carbon fiber (CF) and biochar-modified carbon fiber (BCF) electrodes used as membrane components in MFCs. Four configurations, in duplicate, were constructed by coupling two substrates (biol or FVL) with two membrane types (CF and BCF). All systems exhibited progressive anodic acidification and up to a 55% increase in electrical conductivity. The highest voltage output was achieved in MFC-BL-2 (404.59 mV), followed by MFC-FL-1, driven by synergistic interactions between the substrate and biochar-enhanced conductive networks. MFC-FL-1 also demonstrated superior contaminant removal performance, achieving 60% COD reduction, 36% BOD reduction, and 50% NH_4_^+^–N removal. SEM–EDS analysis confirmed that biochar-modified electrodes developed a porous structure and substantially enhanced microbial adhesion. FVL-fed systems formed dispersed electroactive biofilms that facilitated electron transfer, whereas biol-fed systems developed compact biofilms that constrained electron flux. By integrating waste-derived lignocellulosic materials with electroactive microbial consortia, this work advances a biomimetic circular bioengineering platform for sustainable bioelectrochemical recovery and wastewater remediation.

## 1. Introduction

Global electricity demand increased by 4.3% in 2024, nearly double the growth projected in 2023 [1], underscoring the urgent need to diversify energy sources toward sustainable and resilient alternatives. Although petroleum remains the dominant global energy source worldwide, renewable energy deployment has expanded in regions such as Latin America to provide electricity in remote areas [2]. Hydropower, while widely adopted, can incur significant environmental impacts, including ecosystem disruption and greenhouse gas emissions [3]. In Ecuador, 75.12% of electricity consumption in May 2025 was derived from renewables, predominantly hydropower (73.46%), whereas biomass contributed only 0.72%, declining from 1.65% in 2022 [4]. Simultaneously, global waste generation presents both an environmental challenge and an opportunity for renewable resource recovery. Approximately 998 million tonnes year^−1^ of municipal solid waste are generated worldwide, including 279–300 million tonnes year^−1^ of sugarcane bagasse (SCB) [5]. Ecuador alone produces an estimated 5.26 million tonnes year^−1^ of solid waste, of which 43.9% lacks adequate disposal, and 49.65% corresponds to organic residues [6,7]. Agro-industrial residues, particularly lignocellulosic biomass, constitute abundant renewable feedstocks, with Ecuador generating approximately 2.2 million tonnes annually [8]. The Ecuadorian sugarcane industry recorded a production of 11 million tons in 2021, whereby approximately 42% of processed biomass was categorized as agricultural and industrial residue [7]. SCB, a major by-product of sugar and bioethanol production, possesses high carbon and fermentable fractions, making it an attractive precursor for bioenergy, biofuels, biosorbents, bioplastics, biosensors, and wastewater treatment applications [9,10,11]. Owing to its lignocellulosic composition and porous carbonaceous structure after thermochemical conversion, SCB has also emerged as a promising material for electrochemical and bioelectrochemical systems. Recent studies have demonstrated that SCB-derived biochar can enhance microbial adhesion, electrical conductivity, electroactive surface area, and extracellular electron transfer behavior in microbial fuel cells (MFCs), thereby improving bioelectricity generation and contaminant degradation behavior [12,13]. MFCs are biomimetic bioelectrochemical systems capable of directly converting the chemical energy stored within organic substrates into bioelectricity while simultaneously facilitating wastewater remediation through microbially mediated redox reactions. These systems emulate naturally occurring extracellular electron-transfer mechanisms observed in electroactive microbial ecosystems, where microorganisms interact with conductive surfaces to sustain metabolic energy exchange. In these systems, exoelectrogenic microorganisms oxidize organic substrates at the anode, releasing electrons and protons [14]. Electrons are transferred through an external circuit to the cathode, while protons migrate across the separator membrane (SM) to maintain electrochemical neutrality and complete the electrical circuit [15]. At the cathode, electrons combine with protons and oxygen to produce water [16,17,18]. The electrochemical efficiency of MFCs is strongly governed by substrate composition, membrane characteristics, microbial colonization, and interfacial electron-transfer mechanisms [19]. Substrates with elevated oxidative potential, including SCB hydrolysates, anaerobic digestion effluents (biol), and fruit and vegetable leachates (FVL), can significantly enhance microbial metabolism, biofilm formation, and pollutant degradation, while achieving power densities reaching up to 200 mWm^−2^ [20,21]. In particular, FVL contains a diverse mixture of fermentable sugars, organic acids, amino acids, micronutrients, and biodegradable compounds that support dense, metabolically active electroactive biofilms, thereby facilitating extracellular electron transfer and enhancing anodic activity [22]. Recent advances have further demonstrated that bioelectricity generation and electro-bioremediation of SCB industry effluents can be synergistically optimized by implementing MFCs equipped with activated carbon cathodes [23]. Moreover, the integrated bioelectrochemical conversion of Bacillus subtilis-pretreated SCB revealed that reducing lignocellulosic recalcitrance plays a critical role in enhancing microbial metabolic pathways, coulombic efficiency, and substrate biodegradation in MFC systems [24]. Nevertheless, the majority of existing investigations have primarily focused on the isolated utilization of SCB-derived materials as individual MFC components, including anodic substrates, electron modifiers, cathodic catalysts, or membrane additives [24]. Despite the growing interest in lignocellulosic biomass-derived materials for bioelectrochemical applications, a mechanistic understanding of the coupled interactions among substrate composition, membrane architecture, anodic biofilm development, proton transport behavior, and contaminant removal efficiency remains limited. In particular, systematic comparisons between biochar-modified carbon fiber (BCF) and unmodified carbon fiber (CF) membranes under controlled operational conditions are still scarce. Furthermore, previous studies rarely distinguish whether improvements in MFC performance originate predominantly from substrate-driven microbial activity, membrane-mediated ionic conductivity, or synergistic biofilm-electrode interfacial phenomena. In this context, the present study investigates the integrated utilization of SCB-derived materials within a dual-chamber MFC platform. SCB-derived biochar was employed as a functional modifier for carbon-fiber proton exchange membranes, whereas anaerobic digestion effluent from SCB processing served as the anode substrate. In parallel, FVL was introduced as a nutrient-rich comparative feedstock to evaluate the influence of substrate complexity on microbial electrochemical activity and system performance. To systematically elucidate the interaction between substrate composition and membrane configuration, four reactor systems were constructed under identical operational conditions using partially exposed air cathodes: (i) biol-CF, (ii) biol-BCF, (iii) FVL-CF, and (iv) FVL-BCF. This experimental configuration enabled comparative assessment of voltage generation, electroactive biofilm development, extracellular electron transfer behavior, proton transport characteristics, and contaminant removal efficiency. In addition, microscopic analysis of the membrane surfaces was performed to correlate biochar-induced morphological modifications with microbial attachment and interfacial electrochemical activity. Rather than focusing exclusively on peak electrical output, the study emphasizes the mechanistic relationships among membrane physicochemical properties, substrate biochemical composition, anodic colonization behavior, and bioelectrochemical functionality. Collectively, the integration of lignocellulosic waste valorization, engineered conductive bio-interfaces, and electroactive microbial communities establishes a biomimetic circular bioengineering framework for sustainable energy recovery and environmental remediation.

## 2. Materials and Methods

### 2.1. Materials

Carbon fiber fabric was procured from Carbon Fiber Composites (Houston, TX, USA) and used as both electrode and membrane base material. SCB was collected from a local agro-industrial facility. Analytical-grade acetone, ammonium sulfate ((NH_4_)_2_SO_4_) and sulfuric acid (H_2_SO_4_, 10% *v*/*v*) were purchased from ESPOCH Environmental Chemical Control Department (Riobamba, Ecuador). High-purity nitrogen gas (99.999%, Linde PLC, Dublin, Ireland) was used for pyrolysis under inert conditions. Molasses was sourced from a local supplier. Biol was produced via anaerobic digestion of SCB, while FVL was collected from municipal waste streams in Riobamba, Ecuador. The strains used, *Delftia acidovorans* and *Citrobacter freundii*, were previously isolated, characterized, and stored in the ESPOCH Microorganism Bank. Their taxonomic identity was confirmed through molecular characterization based on 16S rRNA gene sequencing. These strains were selected for the present study due to their reported electroactive behavior, biofilm-forming capability, and potential applicability in bioelectrochemical systems [25,26]. All reagents were analytical grade, and deionized water (Laboratorios ACORSA, Quito, Ecuador) was used throughout.

### 2.2. MFCs Design and Assembly

**Figure 1:**
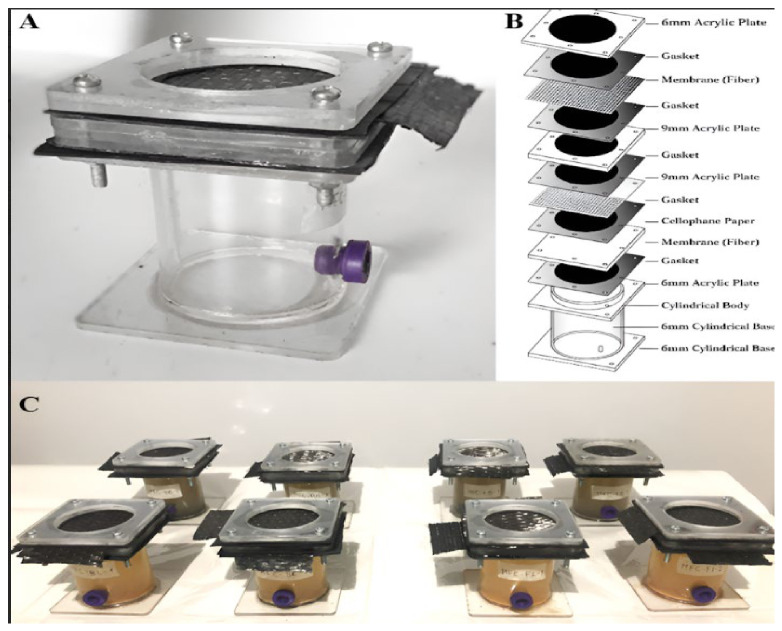
Schematic and photographic depiction of the dual-chamber MFC employed in this study, featuring a cathode partially exposed to ambient air and proton exchange membranes based on carbon fiber (CF) and biochar-modified carbon fiber (BCF). (**A**) Photograph of the assembled MFC reactor; (**B**) schematic illustration of the reactor configuration and electrochemical architecture; (**C**) experimental arrangement of the MFC systems.

Eight dual-chamber MFCs were constructed using acrylic components, each incorporating a cathode partially exposed to ambient air to facilitate oxygen diffusion (Figure 1A). The reactors were fabricated using acrylic plates (6- and 9 mm thickness) to ensure mechanical stability, chemical resistance, and structural integrity under long-term operation. The anodic chamber was cylindrical, with an effective working volume of 250 mL and an internal diameter of 5 cm. A rubber gasket and sealing system were employed between each structural layer to ensure an airtight assembly and to maintain strict anaerobic conditions within the anodic chamber. The anodic and cathodic compartments were tightly secured using galvanized screws, providing stable compression across the membrane–electrode interface, and preventing leakage during operation. Carbon fiber (CF) and biochar-modified carbon fiber (BCF) were used as separator membrane (SM) materials with additional electroactive functionality. Depending on the configuration, CF also served as an electrode material. The membrane–electrode assembly was constructed through a layered stacking strategy, consisting of alternating CF (or BCF) layers, gaskets, and separator sheets to ensure controlled ion transport and stable electrical contact. The complete architecture of the system is illustrated in Figure 1B, which presents an exploded schematic of the MFC configuration, including the acrylic plates, gaskets, membrane layers, and cylindrical reactor body. This modular design enables precise alignment of components, reproducible assembly, and controlled interfacial contact between anodic and cathodic compartments.

The membrane–electrode assembly provided an effective contact area of 19.64 cm^2^ between the anodic and cathodic compartments. The distance between the anodic and cathodic electrodes was 11.3 mm. Oxygen transfer at the cathode occurred via passive diffusion from ambient air, as the cathodic side remained partially exposed to the atmosphere throughout the experimental period, and no external aeration was applied. These operational conditions were kept constant across all MFC configurations.

### 2.3. Experimental Design and Electrode Preparation

The experimental framework followed a full factorial design with two independent variables: (i) separator membrane (SM) composition (pristine carbon fiber, CF, or biochar-modified carbon fiber, BCF) and (ii) substrate (bio-leachate, BL, or fruit and vegetable leachate, FVL) (Table 1).

**Figure 2:**
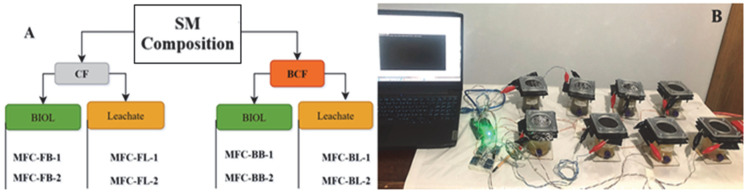
(**A**) Schematic representation of the full-factorial experimental design, illustrating the two-level SM compositions (CF, BCF), two substrate systems (BL and FVL), and duplicate MFC configurations. (**B**) Experimental setup of the MFC systems under operation.

Each treatment combination was performed in duplicate, resulting in eight independent MFCs. The experimental structure is illustrated in Figure 2.

All MFCs employed a unidirectional CF cathode (7 cm × 7 cm), rinsed with distilled water prior to assembly. For BCF-based systems, carbon fiber was modified using sugarcane bagasse ash (SBA), which was dried, milled, sieved, and pyrolyzed under a nitrogen atmosphere from room temperature to 400 °C at 10 °C min^−1^, with 2 h residence time and nitrogen flow of 50 L min^−1^, yielding 28.8–35.15% biochar. The resulting biochar (20 g) was applied to both sides of the CF fabric using a conductive adhesive and mechanically pressed to ensure adhesion. The conductive adhesive served as a binding agent to ensure stable attachment of the biochar particles to the carbon fiber substrate and was not intended to function as an electroactive component. To ensure reproducibility, a thin and uniform layer of adhesive was applied across all biochar-modified electrodes to minimize variability among replicates and maintain consistent electrode preparation conditions. Electrical resistance was measured before and after modification using a digital multimeter under controlled DC conditions. The remaining four MFCs were equipped with pretreated CF anodes. For CF-based systems, untreated CF sheets (7 cm × 7 cm) were chemically pretreated via sequential immersion in acetone (8 h), deionized water rinsing (×5), ammonium sulfate solution ((NH_4_)_2_SO_4_, 50 g in 250 mL), and 10% (*v*/*v*) H_2_SO_4_ treatment for 15 min, followed by drying at 400 °C for 10 min and storage in sealed polyethylene bags.

### 2.4. Substrate Preparation and Inoculation

Two substrates were evaluated across the eight MFC configurations (Table 1 and Figure 2A). Biol was produced via anaerobic digestion of SBA. Briefly, 3.5 kg of SBA (9% moisture content) was homogenized with distilled water, ash, and molasses and processed in a 50 L anaerobic digester operated at a hydraulic retention time of 50 days. Operational parameters, including pH and temperature, were periodically monitored throughout digestion. In parallel, fruit and vegetable leachate (FVL), collected from municipal collected from organic waste streams, was directly used as the comparative substrate. Following substrate preparation, all MFCs were assembled under sterile conditions and inoculated with a mixed bacterial consortium containing 1 mL of *Delftia acidovorans* and 1 mL of *Citrobacter freundii*. These bacterial species are well recognized in environmental and drinking water treatment studies due to their metabolic versatility, environmental adaptability, and strong biofilm-forming capability, which collectively support stable microbial colonization and efficient extracellular electron transfer within MFC systems [27]. In particular, coaggregation between *D. acidovorans* and *C. freundii* may further facilitate biofilm establishment and structural stability, thereby enhancing microbial adhesion, interspecies interactions, and overall electroactive performance within the MFC environment.

### 2.5. Characterization of Substrates and Membrane Materials

Physicochemical and bromatological analyses were performed at the beginning and end of the experimental period to evaluate substrate composition and contaminant removal efficiency. Biochemical oxygen demand (BOD), chemical oxygen demand (COD), and ammoniacal nitrogen (NH_4_^+^–N), pH, electrical conductivity (EC), and temperature were measured according to standard analytical procedures. Nutritional composition, including ether extract (EE), crude fiber (CrF), total protein (TP), nitrogen-free extract (NFE), ash, and moisture content, was determined according to standard AOAC methods. Morphological characterization of CF and BCF membranes was conducted using a scanning electron microscope (SEM; JEOL JSM-IT100, JEOL Ltd., Tokyo, Japan) operated at 15–20 kV with magnifications ranging from 350× to 1400×. Micrographs were obtained from untreated materials and membranes recovered after MFC operation to evaluate surface morphology, biofilm formation, and structural modification.

### 2.6. Operation and Monitoring of MFCs

All MFCs were operated in continuous mode at 20 ± 2 °C. Stable operation was defined by reproducible voltage profiles maintained over at least three consecutive cycles. Voltage output was monitored under open-circuit conditions using an Arduino UNO-based data acquisition system (Arduino LLC, Ivrea, Italy) integrated with an ADS1115 analog-to-digital converter (Texas Instruments, Dallas, TX, USA) (Figure 2). No external resistance or electrical load was applied during voltage monitoring; therefore, the recorded values correspond to open-circuit voltage (OCV). Voltage signals were recorded at 60 s intervals over a 15-day operational period and stored digitally for subsequent analysis.

### 2.7. Structural and Compositional Analysis (SEM-EDX)

Surface morphology and elemental composition of the electrode materials were analyzed using SEM coupled with energy-dispersive X-ray spectroscopy (EDX) (JSM-IT100, JEOL Ltd., Japan). Specimens (10 mm × 10 mm) were mounted on aluminum stubs and sputter-coated with gold prior to analysis. SEM imaging was performed at magnifications of 330×, 430×, and 1800× under an accelerating voltage of 15 kV, using a secondary electron detector to evaluate surface topography and microstructural features. Multiple representative regions were analyzed to ensure reproducibility. Elemental composition was determined by EDX, enabling semi-quantitative analysis of inorganic constituents and mineral distributions across the electrode surfaces. The combined SEM-EDX analysis enabled correlation between surface morphology and compositional heterogeneity of CF and BCF materials.

## 3. Results and Discussion

### 3.1. Control Parameters and Substrate Behavior

**Figure 3:**
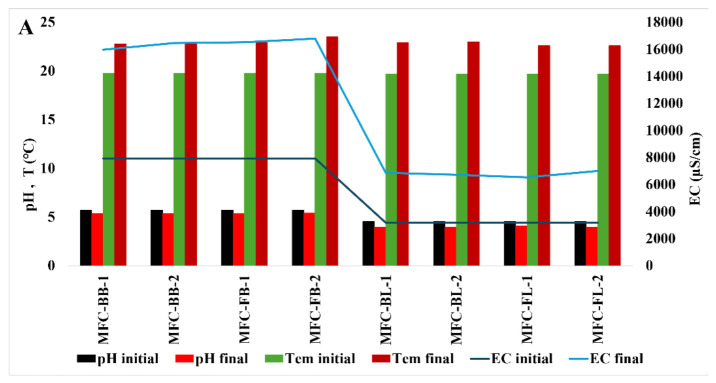

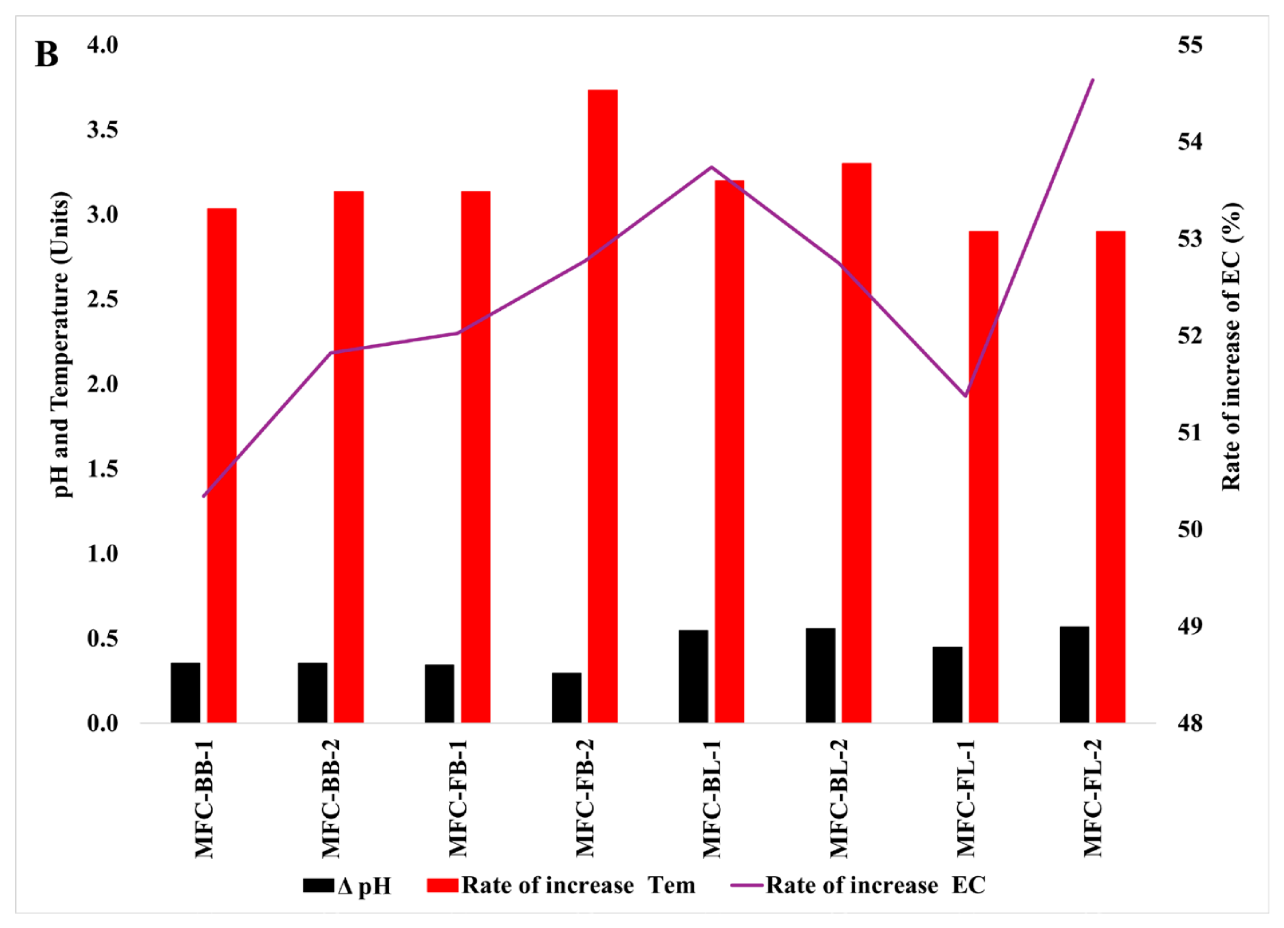
Variations in physicochemical control parameters in MFCs: (**A**) initial and final values of pH, temperature, and electrical conductivity; (**B**) changes (Δ) in pH and temperature and relative increase in EC over the experimental period. Data normality was assessed using the Shapiro–Wilk test (*p* > 0.05), applied in an exploratory manner (ΔT, ΔpH, EC, *p* > 0.05).

The temporal evolution of key physicochemical operating parameters, including pH, temperature (T), and electrical conductivity (EC), was systematically monitored throughout the MFC operation to elucidate the dynamic interplay between substrate transformation, microbial metabolism, and electrochemical activity (Figure 3A). Prior to comparative analysis, data normality was evaluated using the Shapiro–Wilk test (*p* > 0.05) to evaluate the distribution of the measured variables. Given the limited number of replicates, the test was applied in a descriptive and exploratory manner to support data interpretation (Figure 3B). A progressive decline in pH was consistently observed across all configurations, with an average reduction of approximately 0.57 units relative to initial conditions. This acidification of the anodic compartment originates from microbial oxidation of complex organic constituents and the associated proton generation during substrate hydrolysis, fermentation, and metabolic electron release. The effect was more evident in leachate-amended systems, indicating a higher fraction of soluble and readily biodegradable compounds that intensify fermentative and electroactive metabolic pathways. Such pH evolution reflects the continuous biochemical reconfiguration of the anodic environment during substrate mineralization rather than a static acidification process. From a bioelectrochemical standpoint, anodic acidification simultaneously regulates electron-transfer efficiency and microbial stability. Moderate proton accumulation facilitates proton-coupled electron transfer and preserves electrochemical neutrality across the membrane interface, thereby sustaining bioelectrocatalytic activity [28]. Conversely, excessive proton accumulation induces physicochemical stress on electrogenic consortia, adversely affecting biofilm integrity, enzymatic function, and extracellular electron-transfer pathways. Consequently, MFC performance is governed by the dynamic equilibrium between proton production, transport, and microbial adaptation within the anodic microenvironment. This mechanistic interpretation is consistent with reports indicating that optimal MFC functionality is confined to a narrow pH window that maximizes both microbial metabolic activity and interfacial electron-transfer efficiency [29,30,31]. Temperature remained comparatively stable throughout the operational period, fluctuating within ±3.7 °C around the operational set point (20 ± 2 °C). This thermal stability effectively isolates microbial and electrochemical responses from externally induced kinetic perturbations, ensuring that observed variations primarily arise from intrinsic biochemical processes. While enzymatic reaction rates, ion diffusivity, and charge-transfer resistance are inherently temperature-sensitive, the limited fluctuations recorded are insufficient to induce measurable shifts in microbial community function or biofilm performance. Nevertheless, even minor thermal deviations can subtly influence ion mobility and interfacial electrochemical kinetics at the electrode–electrolyte interface, contributing to minor variations in electrical response, as similarly reported for mesophilic MFC systems [32]. EC exhibited a consistent and configuration-dependent increase across all MFC systems, with relative enhancements ranging from 48% to 55%, reflecting continuous ionic enrichment within the anodic compartment during operation. The lowest EC increase was observed in MFC-BB-L, whereas the highest value was recorded in MFC-FVL-2, demonstrating a clear dependence on substrate origin and leachate composition. FVL-based configurations consistently demonstrated higher EC values than BL-derived systems, indicating a greater proportion of readily biodegradable organic fractions and more efficient hydrolytic-fermentative conversion. BL-based systems revealed intermediate EC development due to their mixed composition and more complex degradation pathways, whereas BB-based configurations displayed the lowest EC enhancement, attributable to reduced soluble ionic release and slower mineralization kinetics. This progressive EC increase is directly linked to ongoing biodegradation, mineralization processes, and accumulation of charged metabolic intermediates within the anodic compartment [33,34]. From an electrochemical standpoint, enhanced EC improves electrolyte conductivity, reduces ohmic resistance, and facilitates ion transport, thereby strengthening charge-transfer efficiency across the system [30]. This is particularly evident in the MFC-FVL-2 configuration, where the highest EC increase corresponds to intensified substrate conversion and ionic enrichment. However, despite these favorable conductivity dynamics, the absence of proportionality between EC enhancement and overall treatment performance corroborates that ionic strength alone does not dictate MFC efficiency. Instead, system behavior emerges from the coupled interaction of substrate complexity, microbial community dynamics, mass-transfer limitations, and biofilm conductivity. In particular, FVL-based systems, despite exhibiting higher EC development, may simultaneously promote intensified competitive metabolic pathways that limit direct electron recovery efficiency. Similar decoupling between EC enhancement and treatment performance has been widely reported in MFCs treating heterogeneous lignocellulosic and leachate-derived waste streams [35,36], reinforcing that optimal bioelectrochemical performance depends on a synergistic balance between physicochemical conditions and microbial–electrolyte interactions rather than ionic enrichment alone. The coupled evolution of pH and EC further highlights the progressive reorganization of anodic microenvironments during operation. While increased EC enhances charge-transfer pathways, concurrent acidification imposes physiological constraints on microbial activity, establishing a trade-off between electrochemical conductivity and biological functionality. The stable thermal regime ensures that these coupled effects are not confounded by temperature-induced kinetics variability, confirming that substrate transformation and microbial electroactivity are the dominant drivers of system evolution [37]. These findings collectively emphasize that MFC performance is regulated by interdependent physicochemical feedback mechanisms rather than isolated parameter optimization, highlighting the necessity for integrated operational control under complex substrate matrices [38]. Within this framework, complex substrates provide operational advantages for sustained performance owing to continuous and compositionally diverse organic loading. Unlike readily biodegradable substrates that favor rapid and high instantaneous EC response, leachates containing carbohydrates, proteins, and lipids undergo sequential hydrolysis and fermentation, leading to slower biodegradation kinetics and comparatively reduced coulombic efficiencies due to electron partitioning toward competing non-electrogenic metabolic pathways [39,40].

### 3.2. Nutritional Composition of Substrates

**Figure 4:**
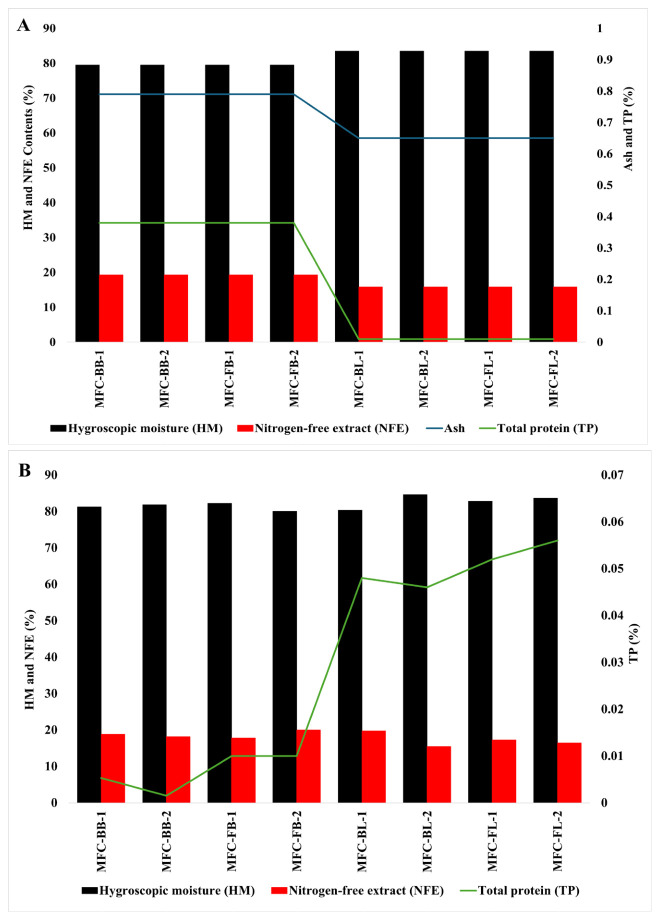
Nutritional composition of the substrates at the beginning and the end of the experimental period: (**A**) initial values; (**B**) final values.

Nutritional characterization of the substrates demonstrated that the hygroscopic moisture (HM) remained the dominant compositional fraction throughout the experimental period, consistently exceeding 70% across all MFC configurations (Figure 4). Among the tested systems, FVL-based configurations generally exhibited slightly higher HM values than BB-derived systems, particularly at the end of the operation, suggesting greater water retention and enhanced solubilization of organic constituents during microbial degradation. In contrast, BB-derived configurations maintained comparatively stable HM values throughout the operational period, indicating more limited substrate transformation under anaerobic conditions. The elevated and stable moisture content across all systems is advantageous for MFC operation because it facilitates ionic mobility, improves substrate diffusivity, and supports microbial colonization within the anodic compartment [41,42]. Nitrogen-free extract (NFE) represented the second-most abundant compositional fraction, remaining within a relatively constrained range of approximately 16–20% across all treatments. Although inter-configuration differences were modest, BL- and FB-derived systems consistently exhibited marginally higher NFE levels compared with BB and FL configurations, while BB systems occupied an intermediate position. These subtle variations likely reflect differences in the accessibility and consumption dynamics of soluble, carbohydrate-rich substrates during MFC operation. Given that NFE is primarily associated with fermentable carbohydrates and other readily biodegradable compounds, its persistence suggests a continuous supply of substrates supporting sustained microbial metabolism and anodic electroactivity. The slight declines observed in several configurations further indicate progressive microbial uptake and conversion of soluble organic during bioelectrochemical processing [43]. Ash content remained comparatively low (<1%) throughout the experimental period and progressively decreased toward the final stage of operation across all configurations. Notably, ash content became undetectable at the end of the cycle, indicating extensive microbial mineralization and substrate transformation during MFC operation [44]. Among the systems, BB-based configurations exhibited slightly higher ash content than BL- and FL-derived systems, likely attributable to greater inherent inorganic content associated with subsurface origin. The overall downward trend in ash-related fractions suggests ongoing mineral transformation, ionic redistribution, and microbial assimilation of inorganic constituents during substrate degradation. Although present in minor quantities, these inorganic fractions may still exert a meaningful influence on local ionic environments. Comparable effects have been reported in MFC systems employing mineral-rich or ash-containing anode substrates, where inorganic phases enhanced microbial colonization and facilitated electron-transfer processes [45]. Total protein (TP) exhibited a pronounced substrate-dependent shift between the initial and final stages of operation, reflecting fundamentally different nitrogen transformation pathways across the MFC configuration. In the BB- and FB-based systems, the initial TP content was relatively high (0.38%) but decreased sharply to trace levels at the final stage (0.0015–0.01%). This substantial reduction indicates intensive protein depletion driven by microbial assimilation, extracellular enzymatic hydrolysis, and subsequent mineralization under prolonged anodic bioelectrochemical activity. The near-complete exhaustion of proteinaceous fractions suggests that these systems promoted efficient utilization of nitrogen-rich components, consistent with strong microbial catabolism and sustained metabolic demand [46,47]. In contrast, the BL- and FL-fed systems demonstrated an opposite trajectory, where initially low TP levels (0.01%) increased markedly over time, reaching 0.046–0.056% at the final stage. This enrichment suggests the accumulation of nitrogen-containing organic compounds within the substrate matrix during MFC operation. Such changes may be associated with microbial growth, metabolic activity, and the formation of soluble or particulate microbial products. However, because TP was measured in the substrate rather than directly on electrode surfaces, the observed increase cannot be interpreted as direct evidence of biofilm development. Therefore, TP variations should be considered an indirect indicator of microbial processing and nitrogen transformation within the system. Collectively, these findings reveal distinct nitrogen processing regimes among the tested substrates and highlight the influence of substrate composition on microbial metabolism during MFC operation [48,49]. Overall, BB and FB configurations were characterized by marked TP depletion, concurrent with relatively stable NFE profiles, indicating preferential protein utilization with limited perturbation of carbohydrate fractions. In contrast, BL and FL systems exhibited TP enrichment coupled with more pronounced NFE variability, reflecting intensified microbial proliferation and more dynamic carbohydrate transformations under leachate-driven conditions. These patterns demonstrate a substrate-dependent coupling between nitrogen assimilation and carbohydrate turnover, underscoring coordinated metabolic reallocation within the MFC systems.

### 3.3. Voltage Generation

**Figure 5:**
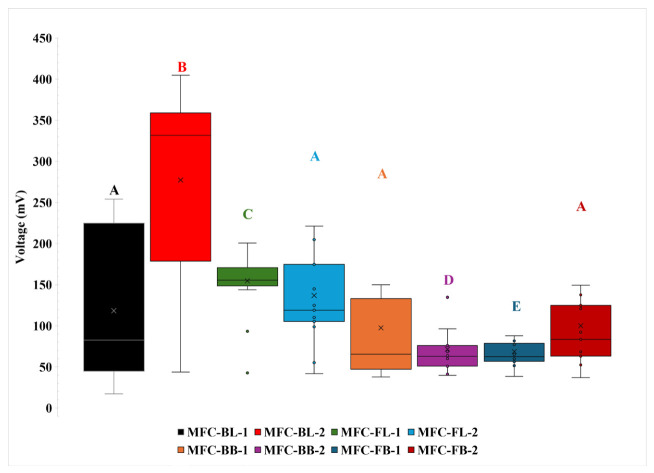
Comparative voltage performance of MFC systems across different substrate types and SM compositions.

Voltage generation varied significantly among the MFC configurations (Kruskal–Wallis, *p* ≤ 0.05), demonstrating that both substrate type and SM composition exert a substantial influence on bioelectrochemical performance (Figure 5). FL-based systems consistently outperformed biol-fed configurations, particularly when integrated with biochar-modified carbon fiber electrodes, as observed in FB- and BB-derived systems. This trend aligns with previous studies reporting enhanced MFC performance in systems utilizing readily biodegradable liquid substrates in combination with conductive carbonaceous electrode materials [50,51], which collectively facilitate microbial colonization, extracellular electron transfer, and charge transport efficiency. Although comprehensive MFC evaluation generally requires the integration of additional electrochemical parameters, including current density, power density, internal resistance, and coulombic efficiency, the present study emphasizes voltage generation as a representative indicator of bioelectrochemical activity, with specific focus on elucidating the effects of substrate characteristics and biochar-modified components on system functionality. Prior to MFC operation, the electrical resistance of the membrane materials was evaluated. Pristine carbon fiber (CF) exhibited a lower electrical resistance (191.80 Ω) than biochar-modified carbon fiber (BCF) (634.14 Ω). Although biochar incorporation increased the measured bulk resistance, the modification introduced a more heterogeneous and porous structure that may facilitate microbial attachment and promote biofilm establishment. Previous studies have reported that biochar-containing carbonaceous materials can enhance electroactive biofilm development and potentially improve interfacial electron-transfer processes through increased surface area, porosity, and biocompatibility, even when variations in bulk electrical resistance are observed. Consequently, the influence of BCF on MFC performance should be interpreted not solely in terms of electrical conductivity but rather as the result of complex physicochemical and biological interactions occurring at the electrode–microorganism interface [22].

The maximum voltage achieved in this study (~404 mV) falls within the typical range reported for laboratory-scale MFCs operating with complex and unrefined lignocellulosic or organic substrates (200–600 mV) [52], though lower than highly optimized systems (>500–700 mV) incorporating engineered electrode and controlled electrochemical conditions [19,37]. The moderate performance may be attributed to the absence of external resistance optimization, limited electrochemical characterization, and insufficient time for full film maturation. Among all configurations, MFC-BL-2 exhibited the highest mean voltage output (227.29 ± 111.70 mV), with peak values reaching 404.59 mV, and displayed a well-defined performance trend among the configurations. This configuration also showed the widest variability, indicative of dynamic electroactive biofilm development and intensified metabolic activity, typically associated with nutrient-rich leachates and high-surface-area electrode materials [53]. Similarly, MFC-FL-1 achieved a relatively high and stable voltage generation (154.75 ± 43.71 mV), further confirming the favorable influence of leachate-based substrates on microbial activity and electron transfer processes. Intermediate performances were observed for MFC-FL-2, MFC-BL-1, MFC-BB-1, and MFC-FB-2, with no significant differences among them, suggesting that while biochar incorporation improves electrode functionality, substrate bioavailability and mass transfer limitations remain key governing factors [49]. In contrast, the lowest voltage outputs were recorded for MFC-BB-2 (71.76 ± 32.71 mV) and MFC-FB-1 (68.63 ± 20.28 mV), both operated with biol. These systems exhibited reduced voltage amplitudes and limited variability, reflecting constrained microbial activity and electron flux. This behavior is consistent with previous findings indicating that digestate-derived substrates typically possess lower soluble organic fractions, slower degradation kinetics, and higher internal resistance within MFC systems [54]. The observed voltage generation profiles exhibited trends consistent with variations in substrate compositional dynamics, particularly TP and NFE fractions (Figure 4). Leachate-based configurations, notably MFC-BL-2 and MFC-FL-1, which demonstrated the highest electrochemical outputs, were characterized by substantial TP enrichment coupled with comparatively dynamic NFE transformation patterns. This behavior suggests intensified microbial proliferation, enhanced extracellular polymeric substance production, and accelerated metabolic turnover resulting from the greater availability of soluble and readily assimilable organic constituents within the leachate matrices. The Progressive increase in TP further reflects active biofilm maturation and biomass accumulation, both of which are intrinsically linked to improved extracellular electron transfer and improved electrochemical performance. Conversely, BB- and FB-derived systems exhibited pronounced TP depletion alongside relatively stable NFE profiles, indicating preferential utilization and exhaustion of proteinaceous fractions with comparatively carbohydrate conversion dynamics. These compositional characteristics corresponded closely with the comparatively lower voltage outputs observed in MFC-BB-2 and MFC-FB-1, suggesting that restricted substrate bioavailability, lower soluble organic content, and reduced metabolic flexibility constrained microbial respiration and electron flux. The relative stability of NFE within these systems may additionally indicate limited accessibility of fermentable carbon fractions, thereby suppressing sustained electroactive microbial activity and reducing overall bioelectrochemical efficiency. Voltage generation followed the order MFC-BL-2 > MFC-FL-1 > MFC-FL-2 > MFC-BL-1 > MFC-FB-2 ≈ MFC-BB-1 > MFC-BB-2 > MFC-FB-1, whereas final TP accumulation adheres the sequence MFC-FL-2 > MFC-FL-1 > MFC-BL-1 > MFC-BL-2 > MFC-FB-1 = MFC-FB-2 > MFC-BB-1 > MFC-FB-2. In contrast, residual NFE was generally higher in FB- and BB-derived systems, following the order MFC-FB-2 > MFC-BL-1 > MFC-BB-1 > MFC-BB-2 > MFC-FB-1 > MFC-FL-1 > MFC-FL-2 > MFC-BL-2. Collectively, these trends indicate that electrochemical performance was more strongly governed by substrate transformation dynamics and TP enrichment than by the persistence of residual carbohydrate fractions. Importantly, this substrate-driven enhancement was further amplified by the incorporation of biochar-modified conductive components, which introduced a distinct structural–electrochemical synergy. It is important to distinguish the relative contributions of substrate composition and electrode modification to the observed electrochemical behavior. In the present study, substrate biodegradability (FL > BL > BB/FB) primarily governed voltage generation through variations in organic matter availability and microbial metabolic activity, whereas biochar modification of carbon fiber electrodes mainly influenced biofilm development and interfacial electron transfer processes rather than serving as the dominant determinant of overall voltage output. The porous architecture and high surface area of biochar likely promoted microbial adhesion and biofilm development, which in turn may have facilitated extracellular electron transfer rather than directly governing overall voltage output. output. When combined with highly biodegradable leachate substrates, these conductive surfaces created a coupled effect in which rapid substrate conversion and efficient electron harvesting mutually reinforced each other. The observed performance trends are consistent with the combined influence of substrate biodegradability and biochar-induced structural modifications, which may have contributed to a more favorable environment for microbial activity and voltage generation, ultimately supporting enhanced interfacial conditions for bioelectrochemical performance [38].

### 3.4. Integrated Contaminant Removal and Energy Recovery from Organic Substrates in MFCs

**Figure 6:**
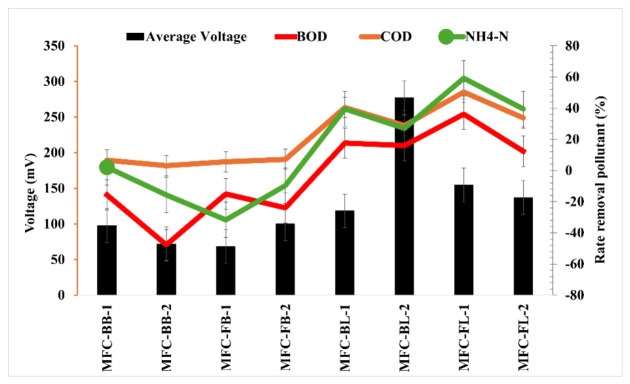
Integrated contaminant removal efficiencies (BOD, COD, and NH_4_^+^–N) and bioelectricity generation profiles of factorially designed MFC systems under different substrate and electrode configurations.

The integrated performance of the MFC systems revealed a tightly coupled but non-linear relationship between contaminant removal, voltage generation, and substrate biochemical dynamics (Figure 6). Overall, chemical oxygen demand (COD) removal consistently exceeded biochemical oxygen demand (BOD) removal across most configurations, while ammoniacal nitrogen (NH_4_^+^–N) exhibited greater variability, reflecting differences in microbial nitrification–denitrification activity and substrate accessibility. Among all systems, MFC-BL-2 and MFL-1 demonstrated the most favorable balance between energy recovery and contaminant removal. In particular, MFC-FL-1 combined high voltage output (~300 mV) with the highest NH_4_^+^–N removal and strong COD reduction, whereas MFC-BL-2 exhibited the highest average voltage (~275 mV) with moderate but consistent pollutant abatement. These results suggest a stronger coupling between organic oxidation and nitrogen transformation processes in FVL-fed systems. In contrast, BB- and FB-derived configurations, particularly MFC-BB-2 and MFC-FB-1, showed significantly lower voltage outputs accompanied by reduced and even negative BOD and NH_4_^+^–N removal efficiencies, suggesting limited substrate biodegradation and possible accumulation of intermediate metabolites. These systems also exhibited lower COD removal, consistent with restricted microbial activity and potentially reduced electron recovery efficiency. Intermediate performance observed in MFC-BB-1 and MFC-FB-2 further reflects partial substrate conversion without efficient electroactive biofilm development. Importantly, the relationship between contaminant removal and voltage generation was non-linear, indicating that pollutant degradation alone does not directly dictate energy recovery. Instead, system performance appears to be governed by the interplay between substrate biodegradability, microbial activity, and biofilm development. FVL-fed systems, enriched in readily assimilable organic fraction, supported more efficient COD reduction and sustained electron flow, whereas Biol-derived configurations were constrained by slower hydrolysis and limited bioavailable carbon fractions [55,56]. Crucially, these electrochemical and removal trends are mechanistically consistent with the previously observed TP-NFE-voltage relationships, providing a unified interpretation of system behavior. Higher-performing configurations (notably MFC-BL-2 and MFC-FB-1) were simultaneously characterized by elevated voltage generation, enhanced TP accumulation, and dynamic NFE turnover, indicating microbial proliferation, robust extracellular polymeric substance formation, and accelerated metabolic flux. In this context, TP enrichment reflects active biofilm maturation and increased electroactive biomass, potentially facilitating enhanced electron transfer, while dynamic NFE utilization indicates efficient conversion of readily biodegradable carbon sources that sustain anodic respiration [57]. Conversely, low-performing systems exhibited reduced TP accumulation and relatively stable or less dynamic NFE profiles, corresponding to constrained microbial growth, limited substrate conversion, and reduced bioelectrochemical activity. These biochemical signatures align closely with their reduced voltage output and inferior contaminant removal performance, confirming that inadequate substrate transformation directly limits both bioelectrochemical activity and pollutant biodegradation [58]. Elevated NH_4_^+^–N concentrations may also have contributed to reduced electrochemical performance in several MFC configurations by adversely affecting ion transport and microbial electron transfer processes. Configurations exhibiting lower NH_4_^+^–N removal, particularly MFC-BB-2 and MFC-FB-1, consistently revealed reduced voltage generation together with limited TP accumulation and relatively stable NFE profiles, indicating constrained microbial metabolism and insufficient substrate conversion. The persistence of ammoniacal nitrogen may lead to ionic imbalances within the system, potentially adversely affecting overall bioelectrochemical performance. In contrast, enhanced NH_4_^+^–N removal observed in the FVL-derived configuration, especially MFC-FL-1 and MFC-BL-2, may have enhanced ionic transport conditions, thereby contributing to the higher voltage outputs observed in these configurations. These findings suggest that NH_4_^+^–N reduction not only improves contaminant removal efficacy but also indirectly boosts MFC performance by alleviating potential ionic transport limitations and promoting conditions conducive to bioelectrochemical activity for sustained bioelectricity generation. Overall, pollutant removal performance differed substantially among the MFC configurations, revealing clear differences in substrate conversion efficiency and microbial electroactivity. Based on the combined behavior of COD, BOD, and NH_4_^+^–N removal together with voltage generation, the overall treatment performance generally followed the order: MFC-FL-1 ≈ MFC-BL-2 > MFC-FL-2 > MFC-FB-2 ≈ MFC-BB-1 > MFC-FB-1 > MFC-BB-2 > MFC-BL-1. Taken together, these results suggest that MFC performance is governed by interconnected relationships among substrate biodegradation (NFE dynamics), biofilm development (TP accumulation), and voltage generation. Within this framework, FVL-based substrates provide a more appropriate environment that simultaneously enhances microbial growth, accelerates substrate conversion, and may support more effective bioelectrochemical activity, ultimately leading to improved integrated wastewater treatment and bioenergy recovery [23].

The performance observed in leachate-fed systems (MFC-BL-2 and MFC-FL-1) aligns with previous studies reporting higher removal efficiencies in substrates rich in readily biodegradable organic matter. Nevertheless, the removal efficiencies obtained in this study (e.g., COD removal up to ~50%) remain lower than those reported in optimized MFC systems, where COD and BOD removal can exceed 80–90% and NH_4_^+^–N removal approaches 70% under controlled conditions [15,59,60]. This discrepancy is likely associated with the relatively short operational period, the lack of system optimization, and the intrinsic complexity of the substrates used, particularly biol. Therefore, these results should be interpreted as evidence of the feasibility of simultaneous bioelectricity generation and partial contaminant removal under non-optimized and realistic experimental conditions.

Additionally, the incorporation of biogenic, carbonaceous biochar-impregnated electrodes was associated with improved contaminant removal and voltage generation. The porous architecture, increased surface area, and enhanced conductivity of the modified electrodes likely promoted microbial adhesion and facilitated extracellular electron transfer, while simultaneously reducing internal resistance. These features may help mitigate the recalcitrance of complex organic substrates and support concurrent pollutant degradation and energy recovery [55,56]. Taken together, these findings highlight the combined influence of substrate type, electrode characteristics, and system configuration on MFC performance. In this context, fruit and vegetable leachates, particularly when coupled with biochar-modified electrodes, represent a promising approach for integrated wastewater treatment and bioenergy generation.

### 3.5. SEM-EDS Characterization of Carbon Fiber and Biochar-Modified Carbon Fiber

**Figure 7:**
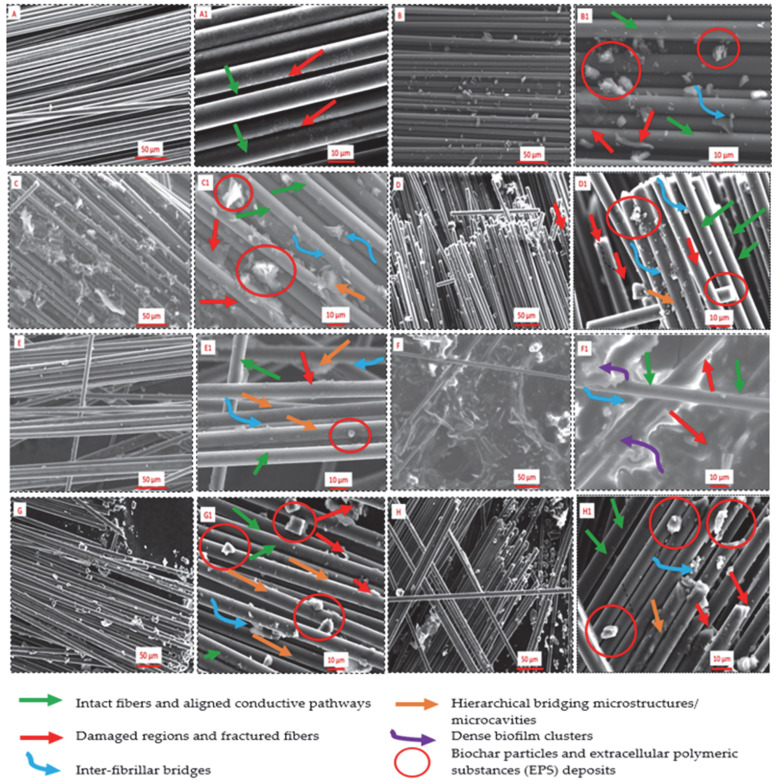
SEM micrographs illustrating the anodic electrode architectures of the MFC configurations: (**A**) pristine carbon fiber, (**B**) biochar-modified carbon fiber, (**C**) MFC-BL-2, (**D**) MFC-FL-1, (**E**) MFC-FB-1, (**F**) MFC-BB-1, (**G**) MFC-BB-2, (**H**) MFC-FB-2. Corresponding high-magnification images (**A1**–**H1**) are provided to further resolve surface morphology and microstructural features. All images include scale bars of 50 µm (low-magnification) and 10 µm (high-magnification). Green arrows indicate intact fibers and aligned conductive pathways; red arrows denote damaged regions and fractured fibers; blue arrows highlight inter-fibrillar bridges; orange arrows indicate hierarchical bridging microstructures/microcavities; purple arrows represent dense biofilm clusters; and red circles mark biochar particles and extracellular polymeric substances (EPS) deposits.

**Figure 8:**
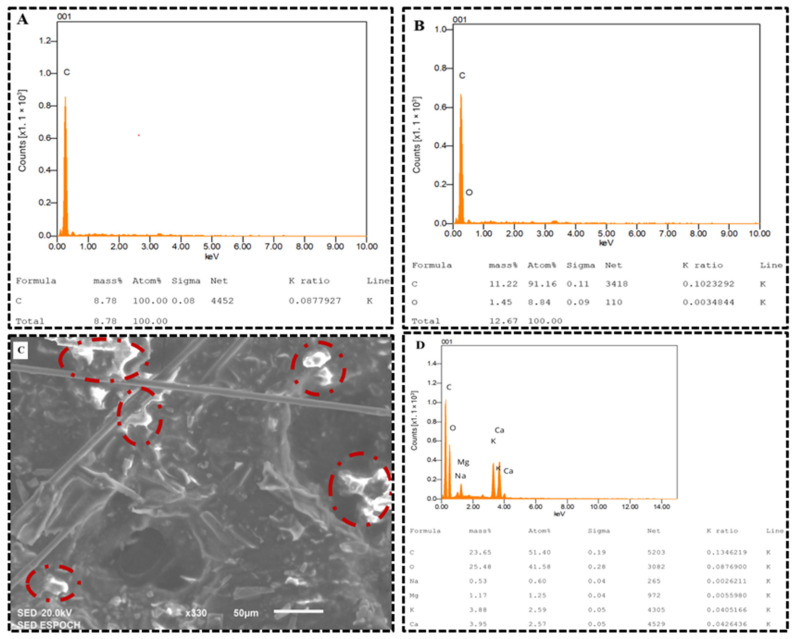
Energy-dispersive X-ray spectroscopy (EDS) spectra and corresponding elemental composition of the anode electrodes: (**A**) carbon fiber (CF) in MFC-FB-2 and (**B**) biochar-modified carbon fiber (BCF) in MFC-BB-1. (**C**) SEM micrograph of the anodic electrode of MFC-BB-2 (×330 magnification; scale bar = 50 µm), showing a heterogeneous surface architecture with localized high-brightness regions (highlighted by red circles) attributed to mineral-enriched or inorganic-rich domains embedded within the carbonaceous matrix, and (**D**) corresponding EDS analysis confirming the elemental composition of the electrode surface.

SEM micrographs coupled with EDS provided detailed insights into the structural and chemical characteristics governing microbial adhesion, biofilm maturation, contaminant transformation, and extracellular electron transfer within the MFC configurations (Figure 7 and Figure 8). Pristine CFs exhibit relatively smooth, compact, and uniformly aligned filamentous surfaces with limited roughness and minimal topographical heterogeneity (Figure 7A). Although these intact conductive pathways favor electron conduction, the comparatively low surface complexity likely restricts microbial anchoring and biofilm nucleation. In contrast, biochar-modified CF (BCF) displayed highly heterogeneous architecture characterized by irregular biochar particulates, porous microdomains, fractured fiber regions, interfiber bridges, and extracellular polymeric substances (EPS)-like deposits (Figure 7B). These hierarchical microstructures likely increased the effective surface area and may have facilitated the formation of conductive micro-networks favorable for microbial attachment and interfacial electron transport [61]. EDS analysis further confirmed the chemically enriched nature of the biochar-modified electrodes (Figure 7 and Figure 8). Carbon remained the dominant elemental constituent across all samples, accompanied by substantial oxygen contributions associated with oxygen-containing functional groups such as hydroxyl, carbonyl, and carboxyl moieties formed through surface oxidation and biogenic carbon deposition during MFC operation. Compared with pristine CF, BCF electrodes exhibited markedly higher elemental heterogeneity, together with detectable inorganic elements, including calcium and potassium, originating from residual biomass minerals and biochar-derived phases and residual biomass constituents. The simultaneous presence of oxygenated functional groups and inorganic mineral species, and conductive carbon frameworks may contribute to a hierarchically structured interfacial environment that influences local surface charge distribution, wettability, and electrochemical reactivity, thereby generating spatially distributed electroactive microdomains that extend beyond purely morphological effects (Figure 7 and Figure 8). These inorganic elements introduce localized high-atomic-number domains that contribute directly to SEM contrast variation, enabling direct visualization of chemically distinct regions across the electrode surface. Correlative analysis between secondary electron imaging and ZAF-corrected EDS spectra confirmed that brighter regions correspond to mineral-enriched domains, whereas darker areas are dominated by carbon–oxygen matrices, verifying that the observed heterogeneity originates from true compositional variations rather than purely topographical effects (Figure 8C,D). The chemically heterogeneous and conductive nature of the biochar-modified surface enhances interfacial functionalities by increasing wettability, surface charge heterogeneity, and electroactive site density [62]. SEM observations are consistent with these interpretations, revealing progressive filling of inter-fiber voids by microbial aggregates, EPS deposits, and biogenic carbon particulates, resulting in localized densification of the electrode matrix and the establishment of interconnected conductive pathways [63]. Following MFC operation, substrate-dependent biofilm morphologies became evident across the electrode surface. Biol-fed systems, particularly MFC-BB-1 and MFC-BB-2, exhibited dense and compact biofilm layers extensively covering both carbon fibers and biochar domains (Figure 7F,G). High-magnification images revealed tightly packed microbial aggregates embedded within EPS matrices and bridging adjacent fibers, indicating strong structural integration between microbial cells and the conductive matrix. However, despite the apparent biofilm density, these configurations demonstrated comparatively lower voltage generation and reduced contaminant removal efficiencies (Figure 6), suggesting that excessive biofilm compactness and partial pore occlusion may have restricted substrate diffusion and mass transfer within the electrode matrix, thereby limiting electroactive functionality [64,65]. Such structural densification can increase mass transport resistance, promote non-uniform current distribution, and ultimately reduce the overall efficiency of electron transport and bioelectrochemical conversion processes within the MFC systems [65]. These observations are consistent with the relatively stable NFE profiles and limited TP enrichment previously explored in these configurations, indicating constrained substrate conversion and lower microbial metabolic activity. Conversely, leachate-fed systems, including MFC-BL-2 and MFC-FL-1, displayed comparatively dispersed yet structurally interconnected biofilm architectures with localized EPS accumulation, porous conductive domains, and partially exposed fiber networks (Figure 7C,D). These configurations corresponded closely with the highest voltage outputs, enhanced TP accumulation, dynamic NFE turnover, and superior effluent treatment performance, such as improved COD and NH_4_^+^–N removal efficiencies. The comparatively heterogeneous and less compact biofilm organization likely facilitated improved substrate diffusion, greater accessibility of electroactive sites, and more efficient proton and electron transport pathways. Simultaneously, the elevated TP levels observed in these systems reflect intensified microbial proliferation and active biofilm maturation, while accelerated NFE depletion indicates efficient utilization of readily biodegradable carbon fractions that sustained respiration and extracellular electron transfer. Importantly, the integrated SEM-EDS observations provide structural and chemical evidence that is consistent with the proposed mechanistic relationships established in the previous sections between substrate biodegradability, TP enrichment, NFE transformation, contaminant removal, and voltage generation. Configurations exhibiting superior electrochemical and effluent treatment performance consistently revealed chemically enriched and hierarchically integrated electrode architectures composed of conductive biochar domains, oxygen-containing functional groups, interconnected microbial clusters, EPS-rich regions, and porous transport pathways. These features collectively appear to promote microbial adhesion, support electroactive biofilm development, and potentially enhance charge transport across the anodic interface. Increased surface roughness and porosity induced by the biochar modification likely provided additional microbial attachment sites and facilitated biofilm establishment, while the heterogeneous carbonaceous domains may have contributed to the formation of conductive pathways. The porous architecture may also have enhanced local mass transport by improving substrate accessibility within the biofilm matrix. However, in the absence of direct electrochemical characterization such as electrochemical impedance spectroscopy (EIS) or cyclic voltammetry (CV), the relative contribution of conductivity enhancement, mass transport improvement, and electron-transfer kinetics cannot be quantitatively distinguished in the present study. Accordingly, the enhanced performance observed in biochar-integrated systems is primarily attributed to the synergistic effects of increased surface roughness, porosity, chemical heterogeneity, and microbial colonization potential [61,66].

Beyond the observed electrochemical improvements, the application of SCB-derived biochar in membrane modification offers promising techno-economic advantages for large-scale bioelectrochemical wastewater treatment applications. SCB is an abundant and low-cost agro-industrial residue widely generated in sugar-producing regions, making it an attractive precursor for the fabrication of functional carbon-based membrane materials. Its incorporation into membrane systems may reduce reliance on more expensive commercial carbon materials while simultaneously promoting waste valorization and circular bioeconomy strategies. Nevertheless, further studies involving techno-economic assessments, membrane durability evaluation, and life-cycle analysis will be required to fully establish the practical feasibility and scalability of biochar-modified membrane systems for industrial wastewater treatment applications.

In the context of further mechanistic refinement, it is acknowledged that advanced electrochemical characterization techniques such as electrochemical impedance spectroscopy (EIS), cyclic voltammetry (CV), polarization analysis, and coulombic efficiency measurements would provide additional depth in elucidating the relationships between electrode microstructure, surface chemistry, and electrochemical behavior performance. Integration of these approaches in future studies will enable more quantitative correlations and a more comprehensive understanding of interfacial processes. In addition, coupling these analyses with microbial community profiling and long-term operational assessments will further strengthen the mechanistic resolution of bioelectrochemical activity within these systems. Within this broader analytical perspective, the present findings establish a robust foundational framework for interpreting the synergistic interactions among electrode microstructure, surface chemistry, substrate transformation, microbial dynamics, contaminant removal, and bioelectricity generation in biochar-integrated MFC systems.

## 4. Conclusions

This study demonstrates that the performance of dual-chamber microbial fuel cells is primarily governed by substrate biodegradability and electrode architecture. The combined use of food–vegetable leachate and sugarcane bagasse-derived biol, in conjunction with carbon fiber (CF) and biochar-modified CF (BCF) membranes, revealed that system behavior is strongly dictated by coupled biochemical and electrochemical processes. Enhanced anodic acidification and increased electrical conductivity (48–55%) indicated intensified substrate biodegradation and improved ionic transport across all configurations. Among the tested systems, the MFC equipped with BCF, and leachate achieved the highest voltage output, whereas the CF-based system with leachate exhibited superior integrated overall contaminant removal efficiency, highlighting the critical role of substrate bioavailability in balancing energy recovery and wastewater treatment performance. SEM-EDS analysis further demonstrated that BCF electrodes provide a structurally and chemically favorable interface resembling naturally occurring porous conductive architecture, thereby facilitating microbial adhesion and extracellular electron transfer. In contrast, dense biofilm formation in biochar-based systems was observed to restrict mass transfer and consequently limit electrochemical performance. Overall, the findings suggest that enhanced MFC performance arises from synergistic interactions between biodegradable substrates and biochar-engineered electrode interfaces. This interplay contributes to improved wastewater remediation and bioelectrochemical energy recovery, while also offering new insights into carbon-based material–biofilm interactions and interfacial electron-transfer processes within nature-inspired circular bioengineering systems.

## Figures and Tables

**Table 1:** Independent variables and factor levels.

Factor	Symbol	Level 1	Level 2	Description
SM composition	P	CF	BCF	Carbon fiber (CF) or biochar-modified carbon fibers (BCF)
Substrate type	S	BL	FVL	Biol (BL) or fruit and vegetable leachate (FVL)
Replication	R	1	2	Duplicate independent reactors

## Data Availability

The data presented in this research are available upon reasonable request from the corresponding author.

## References

[1] IEA Global Energy Review 2025. https://www.iea.org/reports/global-energy-review-2025.

[2] Stringer T., Ramírez-Melgarejo M. (2024). Decarbonization Pathways in Latin America: Assessing the Economic and Policy Implications of Transitioning to Renewable Energy Sources. Next Energy.

[3] Aytac A. (2026). Environmental and Socio-Economic Impact Comparison of Solar and Hydroelectric Systems. Sci. Rep..

[4] ARCONEL Balance Nacional de Energía Eléctrica (BNEE). https://datosabiertos.gob.ec/dataset/https-www-controlrecursosyenergia-gob-ec-balance-nacional-de-energia-electrica.

[5] Bukhari I., Haq F., Kiran M., Aziz T., Mehmood S., Haroon M. (2025). Lignocellulosic Biomass as a Renewable Resource: Driving Second-Generation Biofuel Innovation from Agricultural Waste. Biomass Bioenergy.

[6] Instituto Nacional de Estadística y Censos (INEC), Asociación de Municipalidades Ecuatorianas (AME), Banco de Desarrollo del Ecuador B.P. (BDE) (2024). Estadística de Información Ambiental Económica en Gobiernos Autónomicos Descentralizados Municipales, Gestión de Residuos Sólidos 2023.

[7] Ochoa-Herrera V., Apraez M., Flor D., Flores C., Valencia M., Velasco A. (2025). Comprehensive Analysis of Plastic Regulations in Ecuador: Evaluating the Path to a Circular Economy. Clean. Prod. Lett..

[8] Beluhan S., Mihajlovski K., Šantek B., Ivančić Šantek M. (2023). The Production of Bioethanol from Lignocellulosic Biomass: Pretreatment Methods, Fermentation, and Downstream Processing. Energies.

[9] Kamdem Tamo A., Doench I., Deffo G., Zambou Jiokeng S.L., Doungmo G., Fotsop C.G., Tonleu Temgoua R.C., Montembault A., Serghei A., Njanja E. (2025). Lignocellulosic Biomass and Its Main Structural Polymers as Sustainable Materials for (Bio)Sensing Applications. J. Mater. Chem. A.

[10] Apollon W., Rusyn I., González-Gamboa N., Kuleshova T., Luna-Maldonado A.I., Vidales-Contreras J.A., Kamaraj S.K. (2022). Improvement of Zero Waste Sustainable Recovery Using Microbial Energy Generation Systems: A Comprehensive Review. Sci. Total Environ..

[11] Djafari Petroudy S.R., Rasooly Garmaroody E., Rudi H. (2017). Oriented Cellulose Nanopaper (OCNP) Based on Bagasse Cellulose Nanofibrils. Carbohydr. Polym..

[12] Elakkiya E., Niju S. (2021). Bioelectrochemical Treatment of Real-Field Bagasse-Based Paper Mill Wastewater in Dual-Chambered Microbial Fuel Cell. 3 Biotech.

[13] Bijimol B.I., Elias L., Sreelekshmy B.R., Shibli S.M.A. (2025). Effective Exploitation of Sugarcane Byproducts and Industrial Effluents for Strategic Energy Applications: A Review on Recent Developments and Approaches with Special Reference to Microbial Fuel Cells. ACS Appl. Bio Mater..

[14] Mahmoodi Nasrabadi A. (2026). A Detailed Survey of Microbial Fuel Cells: Classifications, Computational Modeling, Recent Innovations, and Emerging Applications. Renew. Sustain. Energy Rev..

[15] Logan B.E., Hamelers B., Rozendal R., Schröder U., Keller J., Freguia S., Aelterman P., Verstraete W., Rabaey K. (2006). Microbial Fuel Cells: Methodology and Technology. Environ. Sci. Technol..

[16] Greenman J., Mendis B.A., Gajda I., Ieropoulos I.A. (2022). Microbial Fuel Cell Compared to a Chemostat. Chemosphere.

[17] Sato C., Paucar N.E., Chiu S., Mahmud M.Z.I.M., Dudgeon J. (2021). Single-Chamber Microbial Fuel Cell with Multiple Plates of Bamboo Charcoal Anode: Performance Evaluation. Processes.

[18] Guang L., Koomson D.A., Jingyu H., Ewusi-Mensah D., Miwornunyuie N. (2020). Performance of Exoelectrogenic Bacteria Used in Microbial Desalination Cell Technology. Int. J. Environ. Res. Public Health.

[19] Dilip Kumar S., Yasasve M., Karthigadevi G., Aashabharathi M., Subbaiya R., Karmegam N., Govarthanan M. (2022). Efficiency of Microbial Fuel Cells in the Treatment and Energy Recovery from Food Wastes: Trends and Applications—A Review. Chemosphere.

[20] Yoshizu D., Kouzuma A., Watanabe K. (2023). Use of Microbial Fuel Cells for the Treatment of Residue Effluents Discharged from an Anaerobic Digester Treating Food Wastes. Microorganisms.

[21] Obileke K.C., Onyeaka H., Meyer E.L., Nwokolo N. (2021). Microbial Fuel Cells, a Renewable Energy Technology for Bio-Electricity Generation: A Mini-Review. Electrochem. Commun..

[22] Patwardhan S.B., Pandit S., Kumar Gupta P., Kumar Jha N., Rawat J., Joshi H.C., Priya K., Gupta M., Lahiri D., Nag M. (2022). Recent Advances in the Application of Biochar in Microbial Electrochemical Cells. Fuel.

[23] Bose D., Bhattacharya R., Gopinath M., Vijay P., Krishnakumar B. (2023). Bioelectricity Production and Bioremediation from Sugarcane Industry Wastewater Using Microbial Fuel Cells with Activated Carbon Cathodes. Results Eng..

[24] Ratheesh A., Sreelekshmy B.R., Anil Kumar T.R., Sasidharan S., Basheer R., Nair K.S., Nair A.J., Shibli S.M.A. (2025). Integrated Bioelectrochemical Conversion of *Bacillus subtilis*-Pretreated Sugar Cane Bagasse: Metabolic Profile Optimization for Enhanced Microbial Fuel Cell Efficiency and Sustainable Biorefinery Applications. ACS Appl. Bio Mater..

[25] Calderón-Tapia C., Chuquín-Vasco D., Guambo-Galarza A., Núñez-Moreno S., Silva-Cisneros C. (2023). Bioelectricity production from anaerobically treated leachate in microbial fuel cell using *Delftia acidovorans* spp.. AIMS Environ. Sci..

[26] Huang J., Zhu N., Cao Y., Peng Y., Wu P., Dong W. (2014). Exoelectrogenic Bacterium Phylogenetically Related to *Citrobacter freundii*, Isolated from Anodic Biofilm of a Microbial Fuel Cell. Appl. Biochem. Biotechnol..

[27] Afonso A.C., Gomes I.B., Saavedra M.J., Simões L.C., Simões M. (2023). Drinking-Water Isolated *Delftia acidovorans* Selectively Coaggregates with Partner Bacteria and Facilitates Multispecies Biofilm Development. Sci. Total Environ..

[28] Ismail Z.Z., Jaeel A.J. (2013). Sustainable Power Generation in Continuous Flow Microbial Fuel Cell Treating Actual Wastewater: Influence of Biocatalyst Type on Electricity Production. Sci. World J..

[29] Tremouli A., Martinos M., Lyberatos G. (2017). The Effects of Salinity, pH and Temperature on the Performance of a Microbial Fuel Cell. Waste Biomass Valorization.

[30] Raghavulu S.V., Mohan S.V., Goud R.K., Sarma P.N. (2009). Effect of Anodic PH Microenvironment on Microbial Fuel Cell (MFC) Performance in Concurrence with Aerated and Ferricyanide Catholytes. Electrochem. Commun..

[31] Zhang R., Dai W., Xiang H., Chen J., Yi T., Li J., Zhang J., Yang Q., Xiao R., Li X. (2025). Characteristics and Driving Factors of Power Generation Performance in Microbial Fuel Cells: An Analysis Based on the CNKI Database. Front. Microbiol..

[32] Larrosa-Guerrero A., Scott K., Head I.M., Mateo F., Ginesta A., Godinez C. (2010). Effect of Temperature on the Performance of Microbial Fuel Cells. Fuel.

[33] Birjandi N., Younesi H., Ghoreyshi A.A., Rahimnejad M. (2016). Electricity Generation through Degradation of Organic Matters in Medicinal Herbs Wastewater Using Bio-Electro-Fenton System. J. Environ. Manag..

[34] Obata O., Greenman J., Kurt H., Chandran K., Ieropoulos I. (2020). Resilience and Limitations of MFC Anodic Community When Exposed to Antibacterial Agents. Bioelectrochemistry.

[35] Xiao Y., Lin S., Hao T. (2021). Investigating the Response of Electrogenic Metabolism to Salinity in Saline Wastewater Treatment for Optimal Energy Output via Microbial Fuel Cells. Sci. Total Environ..

[36] Cecconet D., Bolognesi S., Piacentini L., Callegari A., Capodaglio A.G. (2021). Bioelectrochemical Greywater Treatment for Non-Potable Reuse and Energy Recovery. Water.

[37] Sonawane J.M., Mahadevan R., Pandey A., Greener J. (2022). Recent Progress in Microbial Fuel Cells Using Substrates from Diverse Sources. Heliyon.

[38] Thakur S., Calay R.K., Mustafa M.Y., Eregno F.E., Patil R.R. (2025). Importance of Substrate Type and Its Constituents on Overall Performance of Microbial Fuel Cells. Curr. Res. Biotechnol..

[39] Zafar H., Peleato N., Roberts D. (2022). A Review of the Role of Pre-Treatment on the Treatment of Food Waste Using Microbial Fuel Cells. Environ. Technol. Rev..

[40] Li S., Chen G. (2018). Effects of Evolving Quality of Landfill Leachate on Microbial Fuel Cell Performance. Waste Manag. Res..

[41] Parwate S.A., Xue W., Koottatep T., Salam A. (2024). Organic Waste for Bioelectricity Generation in Microbial Fuel Cells: Effects of Feed Physicochemical Characteristics. Processes.

[42] Wang C.T., Lee Y.C., Liao F.Y. (2015). Effect of Composting Parameters on the Power Performance of Solid Microbial Fuel Cells. Sustainability.

[43] Scott K., Murano C. (2007). Microbial Fuel Cells Utilising Carbohydrates. J. Chem. Technol. Biotechnol..

[44] Sonu K., Sogani M., Syed Z., Rajvanshi J., Sengupta N. (2025). Performance Evaluation of Microbial Fuel Cell Using Ceramic Anode Blended with Rice Husk Ash and Mild Steel Dust. Sci. Rep..

[45] Sonu K., Sogani M., Maheshwari K., Syed Z., Tiwari M.K. (2025). Steam Pretreatment of Bougainvillea Biomass for Enhanced Bioelectricity Generation and TDS Reduction in Microbial Fuel Cells. Discov. Appl. Sci..

[46] Yadav A., Kumar P., Rawat D., Garg S., Mukherjee P., Farooqi F., Roy A., Sundaram S., Sharma R.S., Mishra V. (2022). Microbial Fuel Cells for Mineralization and Decolorization of Azo Dyes: Recent Advances in Design and Materials. Sci. Total Environ..

[47] Puri L., Hu Y., Naterer G. (2024). Critical Review of the Role of Ash Content and Composition in Biomass Pyrolysis. Front. Fuels.

[48] Srinak N., Chiewchankaset P., Kalapanulak S., Panichnumsin P., Saithong T. (2024). Metabolic Cross-Feeding Interactions Modulate the Dynamic Community Structure in Microbial Fuel Cell under Variable Organic Loading Wastewaters. PLoS Comput. Biol..

[49] Khan S.S., Amjad M., Shareef H., Larkin S. (2024). Review of Microbial Fuel Cell from a Techno-Economic Perspective. Energy Explor. Exploit..

[50] Gezginci M., Uysal Y. (2016). The Effect of Different Substrate Sources Used in Microbial Fuel Cells on Microbial Community. JSM Environ. Sci. Ecol..

[51] Álvarez-Ley J.E., Méndez-Novelo R.I., Giácoman-Vallejos G., Paniagua Solar L.A., San-Pedro L. (2025). Microbial Fuel Cells for Power Generation and Wastewater Treatment: A Review of Components, Performance and Sustainability. Int. J. Hydrogen Energy.

[52] Moqsud M.A., Omine K., Yasufuku N., Hyodo M., Nakata Y. (2013). Microbial Fuel Cell (MFC) for Bioelectricity Generation from Organic Wastes. Waste Manag..

[53] Tesfaye T., Shuka Y., Tadesse S., Eyoel T., Mengesha M. (2025). Improving the Power Production Efficiency of Microbial Fuel Cell by Using Biosynthesized Polyanaline Coated Fe_3_O_4_ as Pencil Graphite Anode Modifier. Sci. Rep..

[54] Kwofie M., Amanful B., Gamor S., Kaku F. (2024). Comprehensive Analysis of Clean Energy Generation Mechanisms in Microbial Fuel Cells. Int. J. Energy Res..

[55] Li S., Cheng C., Thomas A. (2017). Carbon-Based Microbial-Fuel-Cell Electrodes: From Conductive Supports to Active Catalysts. Adv. Mater..

[56] Merga T., Gebreslassie G., Hailu T., Nwanya A.C., Ezema F.I., Ejikeme P.M., Workneh G.A. (2025). Progress of Carbon-Based Electrodes in Microbial Fuel Cells: A Comprehensive Review. Results Chem..

[57] Martin E., Savadogo O., Guiot S.R., Tartakovsky B. (2010). The Influence of Operational Conditions on the Performance of a Microbial Fuel Cell Seeded with Mesophilic Anaerobic Sludge. Biochem. Eng. J..

[58] Karuppiah T., Uthirakrishnan U., Sivakumar S.V., Authilingam S., Arun J., Sivaramakrishnan R., Pugazhendhi A. (2022). Processing of Electroplating Industry Wastewater through Dual Chambered Microbial Fuel Cells (MFC) for Simultaneous Treatment of Wastewater and Green Fuel Production. Int. J. Hydrogen Energy.

[59] Santoro C., Arbizzani C., Erable B., Ieropoulos I. (2017). Microbial fuel cells: From fundamentals to applications. J. Power Sources.

[60] Shabangu K.P., Mthembu N., Chetty M., Bwapwa J.K., Bakare B.F. (2024). A comparative analysis of organic substrates from industrial wastewater streams for enhanced electricity production using a double chamber microbial fuel cell (DCMFC). Energy Rep..

[61] Zhao S., Wang X., Wang Q., Sumpradit T., Khan A., Zhou J., Salama E.S., Li X., Qu J. (2023). Application of Biochar in Microbial Fuel Cells: Characteristic Performances, Electron-Transfer Mechanism, and Environmental and Economic Assessments. Ecotoxicol. Environ. Saf..

[62] Alam J., Ali F.A.A., Alhoshan M., Ghasemi M. (2025). Enhanced Proton Conductivity and Bioenergy Generation in Microbial Fuel Cells Using MXene–PVDF-HFP Nanocomposite Membranes. Energy Fuels.

[63] Meng L., Feng M., Sun J., Wang R., Qu F., Yang C., Guo W. (2022). High-Performance Free-Standing Microbial Fuel Cell Anode Derived from Chinese Date for Enhanced Electron Transfer Rates. Bioresour. Technol..

[64] Yang P., Gao Y., Wang N., Zhu Y., Xue L., Han Y., Liu J., He W., Feng Y. (2023). The Restricted Mass Transfer inside the Anode Pore Channel Affects the Electroactive Biofilms Formation, Community Composition and the Power Production in Microbial Electrochemical Systems. Sci. Total Environ..

[65] Wang N., Liang D., Liu S., Zhang Z., Feng Y. (2026). Revealing the Role of Geobacter in Regulating the Microbial Community during Microbial Electrochemical Systems Operation. J. Environ. Chem. Eng..

[66] Hazzan O.O., Zhao B., Xiao Y. (2023). Strategies for Enhancing Extracellular Electron Transfer in Environmental Biotechnology: A Review. Appl. Sci..

